# Repurposing farnesyltransferase inhibitors tipifarnib and lonafarnib as antibacterial agents: proteomic and functional analyses

**DOI:** 10.3389/fphar.2026.1927718

**Published:** 2026-10-13

**Authors:** Lea Weber, Leila Mokhlessi, Marian Klose, Pascal Dietze, Julia E. Bandow, Sina Schäkermann, Hagen S. Bachmann

**Affiliations:** 1 Institute of Pharmacology and Toxicology, Centre for Biomedical Education and Research (ZBAF), Witten/Herdecke University, Witten, Germany; 2 Applied Microbiology, Ruhr University Bochum, Bochum, Germany

**Keywords:** antibiotics, drug repurposing, farnesyltransferase inhibitors, mode of action, proteomics

## Abstract

Farnesyltransferase inhibitors (FTIs) like tipifarnib and lonafarnib, originally developed as anticancer agents, show antibacterial activity against Gram-positive pathogens through an unknown mechanism of action. Here, we investigated the proteomic response of *Bacillus subtilis* 168 (Gram-positive) and an efflux-deficient *Escherichia coli* Δ*tolC* mutant (Gram-negative) to sublethal concentrations of FTIs. Concentrations resulting in approximately 50% growth inhibition were applied, followed by two complementary proteomic approaches: gel-free quantitative LC-HDMS^E^ (both organisms) and 2D-PAGE with [^35^S]-methionine pulse labelling of newly synthesized proteins (*B. subtilis*). Marker proteins from the *B. subtilis* 2D-PAGE approach were used for comparative proteomic response profiling against a reference library of 96 antibacterial agents. *Bacillus subtilis* was more susceptible to both FTIs than *E. coli* Δ*tolC*, with tipifarnib being more potent. LC-HDMS^E^ analysis revealed a pronounced cell envelope stress signature in *B. subtilis*, including regulation of YocH, YabE, and YjhA, along with several σ^B^, σ^W^, and σ^M^-regulated stress proteins. In the 2D-PAGE analysis, reflecting acute cellular responses by monitoring newly synthesized proteins, both FTIs induced proteins such as ClpC, PspA, and LiaH. Comparative proteomic response analysis in *B. subtilis* showed that both FTIs cluster with membrane-active antibiotics, including xanthoangelol, MP196, gramicidin S, MP66, and nisin, which binds the cell wall precursor lipid II and forms pores in the bacterial membrane. Similarities were also observed with pseudopterosin G and pseudopteroxazole, which target the bacterial cell envelope by integration into the membrane and interfering with membrane-bound steps of cell wall biosynthesis. Yet, the similarity of the proteomic profiles does not indicate a perfect match or an identical molecular mode of action within the reference library. In *E. coli* Δ*tolC*, the proteomic response was less pronounced, but for treatment with tipifarnib, regulated proteins of the category “membrane-bounded periplasmic space” were enriched. Proteomic profiling, combined with functional validation *via* membrane integrity and membrane potential assays, indicates that FTIs compromise the bacterial membrane and activate stress and repair pathways. The FTIs may also indirectly affect cell wall biosynthesis. The lack of a perfect match with any of the 96 compounds in the proteomic reference library highlights FTIs as promising lead structures for the development of novel antibiotics.

## 1 Introduction

Antibiotics are indispensable in modern medicine. They enable effective treatment of bacterial infections, support immunocompromised patients during cancer therapy, and are essential in transplantation medicine as well as postoperative care. However, the increasing prevalence of resistant pathogens threatens these achievements. According to the World Health Organization, antimicrobial resistance accounts for more than 1.2 million deaths annually worldwide (WHO 2023). This urgent situation underscores the need for novel antibiotics with innovative mechanisms of action. One promising strategy to address this challenge is drug repurposing, the application of established compounds for new indications. This approach is widely used in medicine, as it reduces development costs and accelerates the time to clinical use compared to *de novo* drug discovery (Aggarwal et al., 2024; Jampilek, 2022).

An example in this context is the class of farnesyltransferase inhibitors (FTIs). Farnesyltransferase catalyzes a posttranslational modification that is essential for the proper localization and activity of multiple eukaryotic proteins (Fiordalisi et al., 2003; Lane and Beese, 2006). Inhibitors of this enzyme, most prominently tipifarnib and lonafarnib, were originally developed to block oncogenic Ras signaling (Lane and Beese, 2006; Morgillo and Lee, 2006). These FTIs have demonstrated efficacy in other indications, including progeria (Capell et al., 2005) as well as infectious diseases such as hepatitis D (Caviglia and Rizzetto, 2020; Koh et al., 2019), SARS-CoV-2 (Weber et al., 2023), Chagas disease (Kraus et al., 2009), and malaria (Ha et al., 2015). They have further been reported to exert immunomodulatory properties. Studies using the research compound FTI-277 in murine sepsis and endotoxemia showed improved survival, enhanced bacterial clearance, reduced HMGB1 release, attenuation of PD-1/PD-L1-mediated T-cell dysfunction, and protection of mitochondrial function (Yang et al., 2011; Shinozaki et al., 2010; Li et al., 2017; Tsuji et al., 2021). However, as FTI-277 has not advanced to clinical development, it is not suitable for drug repurposing. In contrast, clinically relevant FTIs, i.e., lonafarnib and tipifarnib, may share similar host-directed properties. For example, tipifarnib ameliorated tissue damage in a galactosamine/lipopolysaccharide (LPS) model of acute liver inflammation (Shirozu et al., 2014), while lonafarnib reduced proinflammatory cytokine induction in LPS-stimulated human placental explants (Liong et al., 2018). Furthermore, both compounds attenuated exaggerated inflammatory responses in a cellular model of mevalonate kinase deficiency (Marcuzzi et al., 2011). Together these findings suggest that FTIs may exert beneficial immunomodulatory effects in the context of severe infections, a possibility that warrants further investigation (Li et al., 2017). In addition to these host-directed effects, recent studies have demonstrated that lonafarnib and tipifarnib exhibit direct antibacterial activity. Both FTIs inhibit the growth of Gram-positive pathogens and, when combined with colistin, also affect Gram-negative bacteria (Weber et al., 2021; Klose et al., 2025). In eukaryotic cells, FTIs act *via* inhibition of protein farnesylation. However, bacteria lack this target, suggesting that a different antibacterial mechanism is involved (Weber et al., 2021).

Elucidating this mechanism would not only support the repurposing of FTIs as antibacterial agents but could also provide a basis for the development of novel antibiotics with reduced activity against eukaryotic cells, using FTIs as lead structures. Therefore, the aim of this study was to investigate the antibacterial mode of action of FTIs using complementary proteomic approaches. Initially, we analyzed newly synthesized proteins in *Bacillus subtilis* (Gram-positive) upon FTI treatment using radioactive labeling coupled with 2D-PAGE analysis. This enabled us to assess the acute stress response by monitoring changes in protein biosynthesis over short treatment times and allowed comparison of the proteomic profiles to an existing reference library of 96 antibiotics to derive the mode of action (Senges et al., 2020; Meier et al., 2019; Senges et al., 2022; Janzing et al., 2024). However, as the reference library is specific to *B. subtilis*, we complemented this analysis using a gel-free LC-HDMS^E^-based proteomics approach to monitor long-term adaptation of the proteome in both *B. subtilis* and an efflux-deficient mutant of *Escherichia coli* (Δ*tolC*, Gram-negative). The latter method can be advantageous for monitoring the differential abundance of proteins that are challenging to resolve using 2D-PAGE, such as hydrophobic proteins or proteins with extreme pI values, and can provide insights into adaptation mechanisms based on differential protein abundance and protein stability.

As a meaningful addition to the proteomic approaches, the assessments of membrane integrity using propidium iodide staining, membrane potential using a membrane-potential-sensitive dye, as well as intracellular oxidative changes using fluorescent dyes, provided mechanistic insights into their antibacterial effects.

## 2 Materials and methods

### 2.1 Bacterial strains and chemicals

*Bacillus subtilis* strain 168 was cultivated in Belitsky Minimal Medium (BMM: 15 mM (NH_4_)_2_SO_4_, 8 mM MgSO_4_·7H_2_O, 27 mM KCl, 7 mM Na_3_Citrate·2H_2_O, 50 mM Tris, 0.6 mM KH_2_PO_4_, 2 mM CaCl_2_·2H_2_O, 1 µM FeSO_4_·7H_2_O, 10 µM MnSO_4_·H_2_O, 4.5 mM glutamic acid, 0.78 mM L-tryptophan, 0.2% (w/v) glucose) with constant agitation at 37 °C. *Escherichia coli* BW25113 (wt) and its efflux pump-deficient knockout strain Δ*tolC* from the KEIO collection (Baba et al., 2006) were grown at 37 °C in M9 minimal medium (8.09 mM Na_2_HPO_4_, 12.79 mM KH_2_PO_4_, 8.56 mM NaCl, 18.69 mM NH_4_Cl, 22 mM glucose, 100 μM CaCl_2_, 2 mM MgSO_4_, 29.65 μM thiaminium dichloride, 46.26 μM H_3_BO_3_, 0.01 μM MnCl_2_, 0.77 μM ZnSO_4_, 1.61 μM Na_2_MoO_4_, 0.49 μM CuSO_4_, 0.17 μM Co·(NO_3_)_2_) (Baba et al., 2006).

Tipifarnib and lonafarnib were purchased from SelleckChem (Munich, Germany), and dimethyl sulfoxide (DMSO) from Sigma-Aldrich (St. Louis, MO, United States of America). All compounds were dissolved in DMSO.

### 2.2 Pharmacological characterization of antibiotic effects

#### 2.2.1 Minimum inhibitory concentration (MIC)

The MIC, defined as the lowest concentration preventing visible bacterial growth after 16–18 h of incubation, was determined by serial dilutions in test tubes, each containing 2 mL. The lowest non-turbid concentration was recorded as the MIC. Controls were included to confirm medium sterility and bacterial viability. *Bacillus subtilis* was tested in BMM and *E. coli* Δ*tolC* and *E. coli* wt in M9 medium, each at 5 × 10^5^ cells/mL. Incubations were carried out at 37 °C. Concentrations for both MIC and PEC are listed in Table 1.

**TABLE 1:** MIC and physiologically effective concentrations (PEC) of tipifarnib and lonafarnib in *Escherichia coli* wt, *Escherichia coli* Δ*tolC* and *Bacillus subtilis.*

​	Tipifarnib		Lonafarnib	
	MIC (µg/mL)	PEC (µg/mL)	MIC (µg/mL)	PEC (µg/mL)
*E. coli* wt	>100.0	>100.0	>100.0	>100.0
*E. coli* Δ*tolC*	25.0	12.5	100.0	50.0
*B. subtilis*	10.0	7.5	25.0	12.5

#### 2.2.2 Physiologically effective concentration (PEC)

Cultures were inoculated to an optical density (OD_500_) of 0.05 and grown at 37 °C with shaking (200 rpm). At OD_500_ = 0.35, cultures were split and treated with different concentrations of antibiotics while one culture was left untreated as control. The PEC was defined as the lowest concentration reducing bacterial growth by ∼ 30–80% within the first 2 h of treatment. This concentration ensures that cells experience stress without complete inhibition, thereby inducing adaptive responses on the proteome level.

### 2.3 Gel-free proteomic profiling for *Escherichia coli and Bacillus subtilis*

Quantitative LC-HDMS^E^ analysis was performed for three independent biological replicates. Cultures were grown to OD_500_ = 0.35 and treated with tipifarnib (7.5 μg/mL for *B. subtilis*; 12.5 μg/mL for *E. coli* Δ*tolC*) or lonafarnib (12.5 μg/mL for *B. subtilis*; 50 μg/mL for *E. coli* Δ*tolC*), while controls remained untreated. 10 mL samples were collected 60 min post-treatment. Pellets were washed, resuspended in triethylammonium bicarbonate (TEAB) buffer containing 5% sodium dodecyl sulfate (SDS), and lysed by sonication (VialTweeter; Hielscher, Teltow, Germany). The VialTweeter system was precooled to −20 °C prior to use and kept on ice during operation. Sonication was performed six (for *E. coli* Δ*tolC*) or eight times (for *B. subtilis*), respectively, for 1 min, with a 1-min pause between each cycle. The amplitude was set to 90% with a 0.5 s on/0.5 s off pulse cycle. After centrifugation (21,130 × *g*, 4 °C, 20 min), protein concentrations in the supernatants were determined using the Pierce BCA assay (Thermo Fisher Scientific, Rockford, IL, United States of America).

For MS analysis, 50 µg protein per sample was reduced with 4.5 µL Tris (2-carboxyethyl)phosphine, alkylated with 4.5 µL methyl-methanethiosulfonate (MMTS), acidified with 10 µL 85% phosphoric acid. Samples were diluted with 6-fold excess of binding buffer (100 mM TEAB in 90% methanol, pH 7.55) and loaded onto S-Trap micro columns (Protifi, Fairport, NY, United States of America). Samples were washed three times with 150 µL binding buffer. Digestion was performed with 5 µg trypsin per sample in 20 µL 50 mM TEAB for 2 h at 47 °C. Peptides were eluted in three steps with 40 µL of 50 mM TEAB, 0.2% formic acid (FA), and 50% acetonitrile, respectively. Samples were dried in a vacuum centrifuge and stored at −70 °C until analysis.

Peptides were diluted to 0.2 μg/mL in 3% acetonitrile and 0.1% formic acid. 3 μL of each sample were loaded to a C18 trap column (nanoEase M/Z Symmetry C18 Trap Column, 100 Å, 5 μm, 180 μm × 20 mm, Waters, Milford, MA, United States of America) and separated by ultra-performance liquid chromatography (UPLC, ACQUITY UPLC M-Class System; Waters) on a C18 column (Acquity nanoEase M/Z Peptide BEH C18 Column, 130 Å, 1.7 µm, 75 μm × 20 mm, Waters) at 40 °C and a flow of 0.3 μL/min. The following gradient was used: 0.1% FA in water (solvent A) and 0.1% FA in acetonitrile (solvent B): initial, 1% B; 4 min, 1% B; 7 min, 5% B; 100 min, 40% B; 101 min, 90% B; 104 min, 90% B; 105 min, 1% B, 120 min, 1% B. Eluting peptides were ionized by electrospray ionization and analyzed on a Synapt XS mass spectrometer (Waters) in positive resolution mode, acquiring HDMS^E^ spectra across 50–2000 m/z. Key source parameters: capillary voltage 2 kV; cone voltage 40 V; source temperature 80 °C; desolvation temperature 250 °C; cone gas flow, 50 L/h; desolvation gas flow, 600 L/h. Peptides were fragmented using a collision ramp of 18–47 V and [Glu1-] fibrinopeptide B was fed as lock mass and recorded every 60 s.

Raw data were processed in Progenesis QI for Proteomics (Nonlinear Dynamics, Waters) with the following parameters: ≥2 fragments/peptide, ≥5 fragments/protein, ≥2 peptides/protein, maximum protein size 250 kDa; fixed modification: MMTS (C); variable modifications: oxidation (M), deamidation (N, Q); False discovery rate = 1%. Protein quantification used non-conflicting peptides. Databases: *B. subtilis* 168 (UniProt UP000001570, 4,263 entries) and *E. coli* MG1655 (NCBI NC_000913.3, 4,288 entries) plus PhosB, trypsin, and keratin sequences.

### 2.4 2D gel-based proteomic profiling in *Bacillus subtilis*

Radioactive labeling of newly synthesized proteins and 2D-PAGE profiling were performed in triplicate essentially as described previously (Wenzel et al., 2011). *Bacillus subtilis* cultures were grown to OD_500_ = 0.35 and treated with 7.5 μg/mL tipifarnib, 12.5 μg/mL lonafarnib or left untreated as a control. After 10 min, cultures were pulse-labeled with 1.85 MBq [^35^S]-methionine (Hartmann Analytic, Braunschweig, Germany) for 5 min, and labeling was stopped with chloramphenicol (1 mg/mL). Cells were harvested, washed in TE buffer (100 mM Tris, 1 mM EDTA, pH 7.5), and lysed by VialTweeter sonication as described above. Protein concentrations were determined using a Bradford assay (Roti NanoQuant; Roth, Karlsruhe, Germany). Incorporation of [^35^S]-methionine was quantified by scintillation counting (Tri-Carb 2800TR; PerkinElmer) following precipitation of proteins with 20% trichloroacetic acid.

Proteins were separated by isoelectric focusing (pH 4–7 immobilized pH gradient strips; Cytiva, Marlborough, MA, United States of America) on a Multiphor II system, followed by SDS-PAGE (12.5% acrylamide) using the Ettan DaltTwelve system (Cytiva). Radioactive gels were dried and exposed to phosphor screens (Cytiva) scanned with a Typhoon Trio+ (633 nm excitation, 390 nm emission). Nonradioactive gels were stained with 0.003% ruthenium (II)-tris(4,7-diphenyl-1,10-phenanthroline disulfonate), washed, and scanned at 532 nm excitation/610 nm emission.

Images were analyzed using Delta 2D software (v4.8, Decodon, Greifswald, Germany). Protein regulation factors were calculated from normalized spot intensities relative to controls. Protein spots with ≥ 2-fold increased intensity in all replicates were defined as marker proteins (Senges et al., 2020).

Protein spots were excised, destained (20 mM ammonium bicarbonate in 30% acetonitrile) and digested in-gel with trypsin (6.25 ng/μL for high-intensity, 12.5 ng/μL for low-intensity spots) for 18 h at 37 °C. Peptides were eluted in 0.1% trifluoroacetic acid by sonication and analyzed *via* UPLC-MS^E^ on a Vion IMS QToF mass spectrometer (Waters) using electrospray ionization in positive sensitivity mode. 5 μL of each sample were separated on an Acquity UPLC-BEH C18 column (2.1 mm × 50 mm, particle size 1.7 µm) at a flow rate of 0.4 mL/min and the following gradient using solvents A and B as described above: initial, 1% B; 0.5 min, 1% B; 4.75 min, 40% B; 5 min, 85% B; 5.5 min, 85% B; 5.6 min, 1% B; 7 min, 1% B. Spectra were recorded in a range of 50–2000 m/z with a scan time of 0.2 s using the following parameters: capillary voltage, 0.8 kV; cone voltage, 40 V; source temperature, 150 °C; desolvation temperature, 550 °C; cone gas flow, 50 L/h; desolvation gas flow, 1000 L/h. For fragmentation a collision ramp from 28 to 60 V was used and leucine enkephalin was recorded as lock mass every 5 min. Data were exported from UNIFI (Waters, v1.9.13) to waters raw format and processed in ProteinLynx Global Server (Waters, v3.5.3) with the following parameters: lock mass window 0.25 Da; low/high energy thresholds 50/10 counts. Proteins were identified against a non-redundant *B. subtilis* 168 database (Uniprot ID: UP000001570) plus trypsin and keratin sequences, using criteria of ≥2 fragment matches/peptide, ≥5 matches/protein, ≥2 peptides/protein, maximum mass 250 kDa, trypsin digestion (1 missed cleavage), carbamidomethyl (C) fixed, deamidation (N, Q) and oxidation (M) variable; false discovery rate 4%.

### 2.5 Comparative proteomic response (CoPR) analysis

Marker protein profiles were compared to the CoPR reference library of 96 compounds using cosine similarity scoring as described previously (Senges et al., 2020). Analysis was performed using a Python script available online (Yayci et al., 2021). A score of 1.0 indicates identical proteomic response profiles and a score of 0 indicates no similarity.

### 2.6 Membrane integrity assessment

Membrane integrity was assessed using LIVE/DEAD *Bac*Light Bacterial Viability Kit (Invitrogen, Carlsbad, CA, United States of America). Overnight cultures were diluted 1:200 into fresh Lysogeny Broth and grown to mid-log phase. Cell density was normalized to OD_500_ = 0.3, washed, and treated for 1 h. Samples were washed, stained with SYTO9 and propidium iodide (PI) for 15 min in the dark according to the manufacturer’s protocol, washed again, and imaged by fluorescence microscopy. Fluorescence microscopy was performed using a KEYENCE BZ-X810 Fluorescence Microscope (KEYENCE Corporation, Osaka, Japan) equipped with a PlanApo ×40 dry objective (NA 0.95). SYTO9 and propidium iodide fluorescence were detected using GFP (Ex 470/40 nm, Em 525/50 nm) and TRITC (Ex 545/25 nm, Em 605/70 nm) filter sets, respectively. Single-plane images were acquired using automatic exposure settings in BZ-X800 Viewer software (version 1.3.0.1). Representative fluorescence images were processed using Fiji v2.16.0/ImageJ v1.54p (Schindelin et al., 2012), and the same linear contrast adjustment was applied across all treatments. Images were used for the qualitative visualization of SYTO9-stained and PI-positive bacteria; fluorescence intensities were not quantified. For each condition, at least 100 cells from three independent experiments were evaluated to determine the proportion of PI-positive cells.

### 2.7 Membrane potential assay

The membrane-potential-sensitive dye 3,3′-diethyloxacarbocyanine iodide (DiOC_2_ (Fiordalisi et al., 2003)) (Thermo Fisher Scientific) was used to measure changes in membrane potential and the assay was performed according to Zhang *et al.* (Zhang et al., 2022). This lipophilic cationic fluorescent dye can be used to assess changes in transmembrane potential (ΔΨ) indicating early-stage membrane permeabilization prior to complete structural rupture. Red fluorescence reflects the accumulation and aggregation of the dye in polarized cells. Green fluorescence indicates that the dye remains in its monomeric form, so that a decrease of this red-to-green fluorescence ratio reveals membrane depolarization (Gentry et al., 2010). Bacterial cultures were grown in LB (+0.5% glucose for *B. subtilis*) to mid-logarithmic phase (OD_600_ of 0.35). Suspensions in phosphate-buffered saline (PBS) were prepared from harvested cell pellets, followed by staining with DiOC_2_ (Fiordalisi et al., 2003) at a final concentration of 30 µM for 15 min at 37 °C (180 rpm shaking, protected from light). After transferring the stained cell suspension into black, polystyrene 96-well microtiter plates, the fluorescence signal was monitored until reaching a stable baseline using an Infinite M Plex microplate reader. The reaction was started by adding the compounds to be tested and fluorescence (excitation: 485 nm, emission for green fluorescence: 530 nm; emission for red fluorescence: 630 nm) was measured every 2 min for 30 min. Compounds were added in the following concentrations: tipifarnib, 7.5 and 10 μg/mL (*B. subtilis*) or 12.5 and 25 μg/mL (*E. coli* Δ*tolC*); lonafarnib, 12.5 and 25 μg/mL (*B. subtilis*) or 50 and 100 μg/mL (*E. coli* Δ*tolC*); DMSO (1%). Nisin (20 μg/mL) and carbonyl cyanide 3-chlorophenylhydrazone CCCP (5 µM) served as positive controls for *B. subtilis*, while colistin (2 μg/mL) and CCCP (5 µM) served as controls for *E. coli* Δ*tolC*.

### 2.8 Measurement of the intracellular redox state

The intracellular redox state was assessed using 2′,7′-dichlorodihydrofluorescein diacetate (DCFH-DA) (Sigma Aldrich, St. Louis, MO, United States of America). This non-fluorescent dye is cell-permeable and undergoes intracellular deacetylation to DCFH (Wang and Joseph, 1999). Bacteria were grown in LB to an OD_600_ of 0.35. Cells were subsequently treated with the following compounds: tipifarnib, 7.5 and 10 μg/mL (*B. subtilis*) or 12.5 and 25 μg/mL (*E. coli* Δ*tolC*); lonafarnib, 12.5 and 25 μg/mL (*B. subtilis*) or 50 and 100 μg/mL (*E. coli* Δ*tolC*) or DMSO (1%) for 1 h at 37 °C and 180 rpm. After the treatment, cells were centrifuged (5,000 x *g*, 5 min), washed three times with PBS, and resuspended in PBS. The OD was normalized to ∼0.35. Samples were then incubated with 5 µM DCFH-DA for 30 min at 37 °C in the dark under continuous shaking at 180 rpm. For the measurement, the suspensions were transferred to black 96 well polystyrene microtiter plates and fluorescence was measured using an Infinite M Plex microplate reader (Excitation: 485 nm, Emission: 530 nm).

### 2.9 Statistics

Experiments were performed as biological triplicates. For the gel-free proteomic profiling, proteins were only considered to be significantly regulated when they showed a p-value of less than 0.05 and a log_2_ fold change outside the respective 95% confidence intervals. For the statistical analysis of the membrane integrity assay, one-way ANOVA followed by Dunnett’s *post hoc* test was performed. Statistical analyses were performed for the measurements of the intracellular redox state using a two-way repeated-measures ANOVA with Dunnett’s *post hoc* test for multiple comparisons.

## 3 Results

### 3.1 Susceptibility of *Escherichia coli* Δ*tolC* and *Bacillus subtilis* to FTIs

Susceptibility testing revealed a pronounced strain-specific difference in response to the FTIs tipifarnib and lonafarnib (Table 1). Both compounds showed no MIC against *E. coli* wt up to the highest tested concentration of 100 μg/mL. The MICs for the efflux-deficient strain *E. coli* Δ*tolC* were 25 μg/mL for tipifarnib and 100 μg/mL for lonafarnib, respectively. MICs for *B. subtilis* were two-to four-fold lower than those observed in *E. coli* Δ*tolC*. In order to stress the cells for proteomic profiling, concentrations leading to a visible growth inhibition without abolishing growth completely, were determined. These physiologically effective concentrations (PEC) determined for *E. coli* Δ*tolC* were 12.5 μg/mL for tipifarnib and 50.0 μg/mL for lonafarnib, and 7.5 μg/mL for tipifarnib and 12.5 μg/mL for lonafarnib in *B. subtilis*. Thus, across both species, tipifarnib consistently demonstrated lower MICs and physiologically effective concentrations than lonafarnib.

### 3.2 Newly synthesized proteins reveal acute cell envelope stress in *Bacillus subtilis*

The acute stress response was analyzed by 2D-PAGE of newly synthesized proteins labeled with [^35^S]-methionine in *B. subtilis* (Figure 1). This approach allows comparison of the acute proteomic response after treatment with tipifarnib and lonafarnib for 15 min with an extensive proteomic reference library to globally assess the mode of action (Senges et al., 2020; Meier et al., 2019; Senges et al., 2022; Janzing et al., 2024). As expected, treatment with FTIs resulted in methionine incorporation levels comparable to the control (Figure 1B), indicating that global protein biosynthesis was not substantially affected.

**FIGURE 1:**
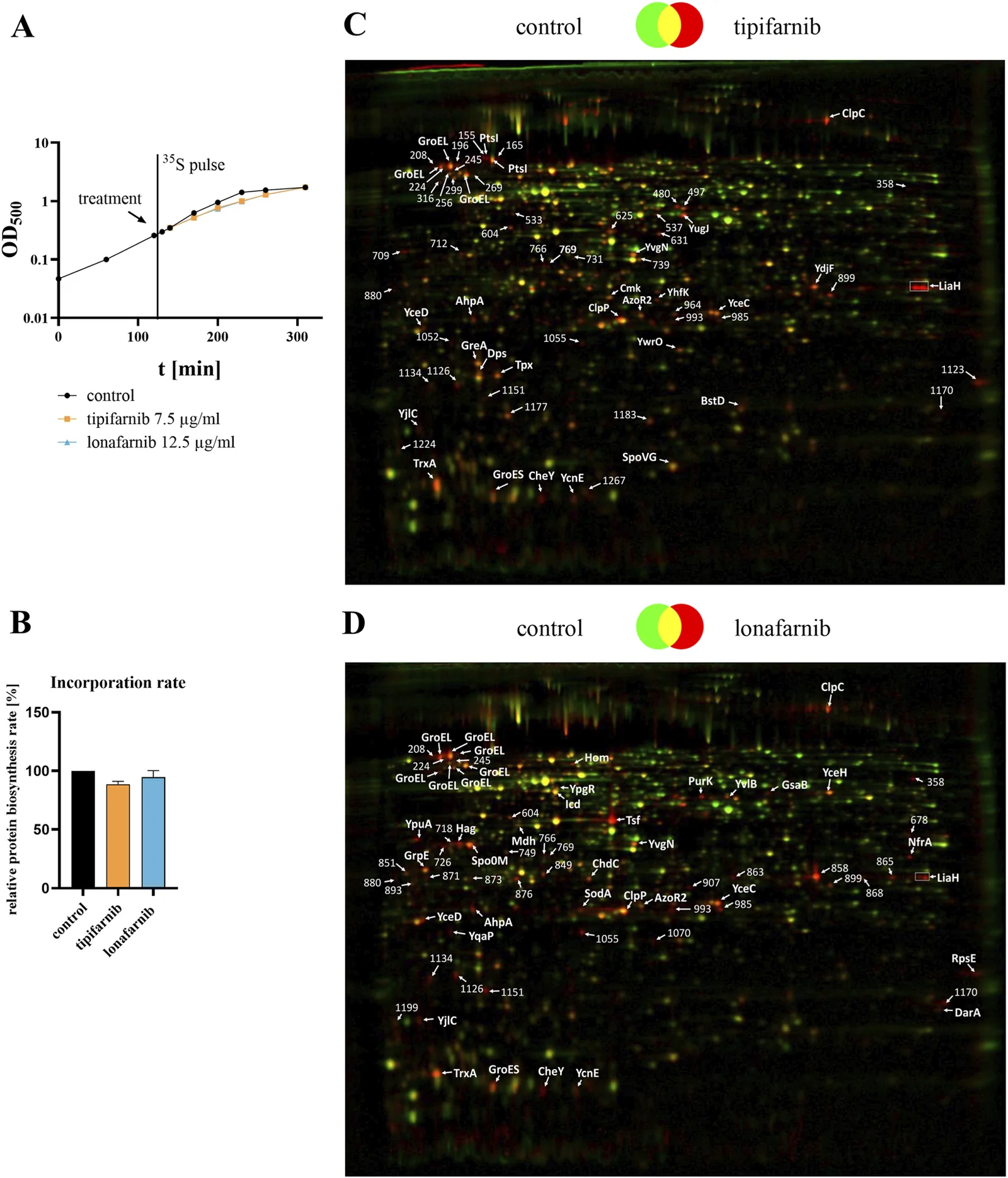
2D-PAGE analysis of newly synthesized proteins in *Bacillus subtilis* after treatment with farnesyltransferase inhibitors. Early exponential phase cultures were treated with physiologically effective concentrations (PECs) of tipifarnib or lonafarnib for 15 min. **(A)** Growth curves of tipifarnib or lonafarnib-treated cultures as well as untreated controls. Data representative of three biological replicates are shown. **(B)** Relative incorporation rates of [^35^S]-methionine during a 5 min pulse labeling under the same conditions (means ± SD, n = 3). **(C,D)** Images of autoradiographs of 2D-PAGE gels showing newly synthesized proteins after tipifarnib **(C)** or lonafarnib **(D)** treatment (red) were overlayed with respective gel images of untreated controls (green). Upregulated proteins appear red, downregulated ones green, and proteins with unchanged relative synthesis rates appear yellow. Marker proteins with ≥2-fold increased relative intensity compared to the untreated control in all three replicates are indicated. Spot identities were determined by LC-MS^E^. Numbers indicate proteins that could not be identified.

In total, 68 protein spots upon tipifarnib treatment and 76 protein spots upon lonafarnib treatment exhibited a two-fold increase in signal intensities in all three biological replicates (Figure 1). Mass spectrometric analysis enabled the identification of marker proteins in 26 of 68 spots for tipifarnib (Figure 1C) and 33 of 76 spots for lonafarnib (Figure 1D). The remaining spots were visible on autoradiographs, but protein quantities were insufficient for detection by fluorescence staining in preparative gels. A comprehensive overview of these marker proteins, including induction factors, MS-based identification, functional annotation, and known regulators, is provided in Table 2. The most prominent groups comprised proteins associated with cell envelope stress and the general stress response, such as LiaH, PspA, YceC, and YceD, which were induced upon treatment with both FTIs. In addition, lonafarnib treatment upregulated the proteins YpuA, YvlB, and YceH.

**TABLE 2:** Marker proteins identified *via* 2D gel-based proteomic analysis after FTI treatment in *Bacillus subtilis.* Proteins were categorized according to the Subtiwiki gene categories (level 2) (Elfmann et al., 2025). NR: not regulated. Proteins highlighted in bold were regulated for both tipifarnib and lonafarnib. For proteins with induction factors written in italics, the protein was also found regulated in the respective gel-free proteomic analysis.

Marker protein	Induction factor		Protein function	Regulon	Category
	Tipifarnib 7.5 μg/mL	Lonafarnib 12.5 μg/mL			
**ClpC**	**3.54 ± 3.26**	**3.90 ± 1.72**	AAA unfoldase	σ^M^, CtsR, σ^B^, σ^F^, Spx	Cell envelope and cell division
**PspA**	**3.28 ± 2.19**	**20.88 ± 8.25**	Phage shock protein a homolog	σ^W^, AbrB	Cell envelope
YpuA	NR	*6.55 ± 4.90*	Unknown	σ^M^	Cell envelope
YvlB	NR	4.11 ± 2.12	Unknown	σ^W^	Cell envelope
**LiaH**	**15.65 ± 13.79**	**8.43 ± 2.65**	Similar to phage shock protein	LiaR	Cell envelope
SpoVG	2.29 ± 0.35	NR	RNA-binding regulatory protein	AbrB, SinR, σ^H^	Cell division
**YceD**	**3.29 ± 0.72**	***5.10 ± 1.92***	General stress protein	σ^M^, σ^W^, σ^X^, σ^B^	General stress
YceH	NR	5.01 ± 1.52	Similar to toxic anion resistance protein	σ^M^, σ^W^, σ^X^, σ^B^	General stress
SodA	NR	*3.98 ± 1.52*	Superoxide dismutase	σ^B^	General stress
**YceC**	***3.46 ± 0.62***	***9.24 ± 2.94***	Similar to tellurium resistance protein	σ^M^, σ^W^, σ^X^, σ^B^	General stress
**YvgN**	***5.39 ± 0.39***	***3.89 ± 0.54***	Glyoxal reductase	σ^B^, SinR	General stress
YhfK	3.93 ± 1.48	NR	Ene reductase	​	Coping with stress
Tpx	3.32 ± 0.18	NR	Thiol peroxidase	Spx	Coping with stress
YugJ	*6.46 ± 1.77*	NR	NADPH-dependent furan aldehyde reductase	Spx	Coping with stress
***TrxA***	***3.42 ± 0.21***	***3.11 ± 0.38***	Thioredoxin	CtsR, σ^B^,Spx	Coping with stress
**ClpP**	**3.84 ± 1.00**	**4.64 ± 1.18**	ATP-dependent Clp protease proteolytic subunit	σ^B^, CtsR	Protein synthesis
**GroEL**	**5.74 ± 5.09**	**13.36 ± 16.56**	Chaperonin and co-repressor for HrcA	HrcA	Protein synthesis
**GroES**	**3.02 ± 0.40**	**3.63 ± 0.20**	Chaperonin	HrcA	Protein synthesis
GrpE	ND	3.42 ± 0.69	Heat-shock protein	HrcA	Protein synthesis
**RpsE**	**6.78 ± 1.24**	**5.72 ± 1.64**	Ribosomal protein S5	​	Protein synthesis
**Tsf**	**2.51 ± 0.42**	**7.70 ± 5.54**	Elongation factor Ts	​	Protein synthesis
GreA	2.92 ± 0.61	NR	Transcription elongation factor	​	RNA synthesis
**CheY**	**9.90 ± 2.13**	**8.98 ± 2.46**	Two-component response regulator	Spo0A, SwrA, σ^D^, CodY, DegU	Regulation of gene expression
Hom	NR	3.89 ± 1.02	Homoserine dehydrogenase (NADPH)	CodY, TnrA, ThrR	Amino acid/N metabolism
Mdh	NR	2.15 ± 0.16	Malate dehydrogenase	CcpA, CcpA, CitB	Carbon metabolism
Icd	NR	2.30 ± 0.28	Isocitrate dehydrogenase	CcpA, CcpC, CitB	Carbon metabolism
PurK	NR	8.23 ± 3.45	Phosphoribosylamino-imidazole carboxylase	G-box, PurR	Nucleotide metabolism
Cmk	2.29 ± 0.18	NR	Cytidylate kinase	​	Nucleotide metabolism
GsaB	NR	3.98 ± 1.10	Glutamate-1-semialdehyde aminotransferase	​	Other pathways
Dps	5.87 ± 5.68	NR	Iron storage protein	σ^B^	Other pathways
ChdC	NR	2.59 ± 1.04	Coproheme decarboxylase	​	Other pathways
YwrO	3.61 ± 0.38	NR	Similar to NAD(P)H oxidoreductase	​	Electron transport and ATP synthesis
**Hag**	**2.80 ± 0.39**	**11.60 ± 1.56**	Flagellin protein	σ^D^, CodY, CsrA, ScoC	Motility and chemotaxis
**AzoR2**	**2.27 ± 0.05**	**2.75 ± 0.47**	Similar to NAD(P)H dehydrogenase	σ^B^, MhqR	Sporulation
Spo0M	NR	*6.98 ± 0.73*	Sporulation-control gene	σ^W^, σ^H^	Sporulation
**YcnE**	**3.01 ± 0.40**	**2.84 ± 0.53**	Putative monooxygenase	​	Phosphoproteins
**YqaP**	**5.77 ± 1.21**	**10.34 ± 3.20**	Unknown	AbrB	Prophages
PtsI	138.64 ± 193.83	NR	Phosphotransferase system enzyme I	GlcT	Transporters
DarA	NR	6.99 ± 1.93	c-di-AMP-binding PII-like protein	​	Unknown
**YjlC**	**3.75 ± 0.34**	**5.53 ± 2.85**	Uncharacterized protein	ResD, Rex	Unknown
YpgR	NR	3.02 ± 0.97	Uncharacterized protein	​	Unknown
BstD	*3.22 ± 0.90*	NR	Bacillithiol S-transferase	​	Unknown
**AhpA**	**4.49 ± 0.79**	**6.58 ± 2.30**	Alkyl hydroperoxide reductase	AbrB	Unknown

Many marker proteins detected under both treatments were involved in the σ^B^-mediated general stress response, including YceC, YceD, and YvgN. Several proteins detected were associated with oxidative stress protection. Specifically, tipifarnib induced the expression of Tpx, YugJ, YwrO, and TrxA, whereas lonafarnib upregulated, e.g., SodA, TrxA, and BstD.

Another major group induced by both FTIs comprised chaperones and proteases (ClpC, ClpP, GroEL, and GroES) involved in protein quality control. These, along with RpsE and Tsf, all play essential roles in the protein life cycle. Additionally, proteins involved in amino acid and nucleotide metabolism, e.g., Hom and PurK (lonafarnib) and Cmk (tipifarnib) were identified.

Taken together, both tipifarnib and lonafarnib rapidly induced cell envelope stress proteins, proteins of the general stress response, as well as proteins of the proteotoxic stress response, specifically to counteract the misfolding of proteins (Table 2).

### 3.3 Mode of action profiling links FTIs to membrane-active antibiotics

The proteomic profiles of tipifarnib and lonafarnib were compared to profiles previously recorded for 96 antibiotic agents to derive their mode of action (Senges et al., 2020; Meier et al., 2019; Senges et al., 2022; Janzing et al., 2024). Similarity of proteomic profiles was assessed mathematically, resulting in cosine similarity scores as output (1 = perfect similarity, 0 = perfect dissimilarity) that were visualized in a tSNE plot. The similarity between the two FTIs was highest with 0.52 (Figure 2; Supplementary Table S1), indicating that they share the mode of action. All other reference compounds showed lower similarity scores (Figure 2; Supplementary Table S1). This indicates that the proteomic response profiles of FTIs did not fully correspond to any of the 96 reference compounds. Both FTIs did not show significant similarity to antibiotics targeting RNA polymerase (e.g., rifampicin), DNA gyrase (e.g., ciprofloxacin), protein biosynthesis inhibitors or compounds having effects on redox homeostasis, metal homeostasis or nucleic acids. Yet, the proteomic profiles of tipifarnib and lonafarnib showed similarity to compounds interfering with membrane structure or membrane-associated processes. For example, the similarity score of tipifarnib was highest to the geranylated hydroxy chalcone xanthoangelol (0.35), the pseudopterosin G (PsG, 0.32), and the antimicrobial peptides MP66(L) (0.31), and gramicidin S (0.30). Additionally, the proteomic profile of both compounds shared similarity to nisin (0.29 for tipifarnib, 0.30 for lonafarnib), which binds to the cell wall precursor lipid II and is known to form membrane pores. Lonafarnib shared the highest scores with the antimicrobial peptides MP196-C-C0 and MP196(L), as well as xanthoangelol (0.44, 0.43, and 0.40, respectively) (Figures 2B,C).

**FIGURE 2:**
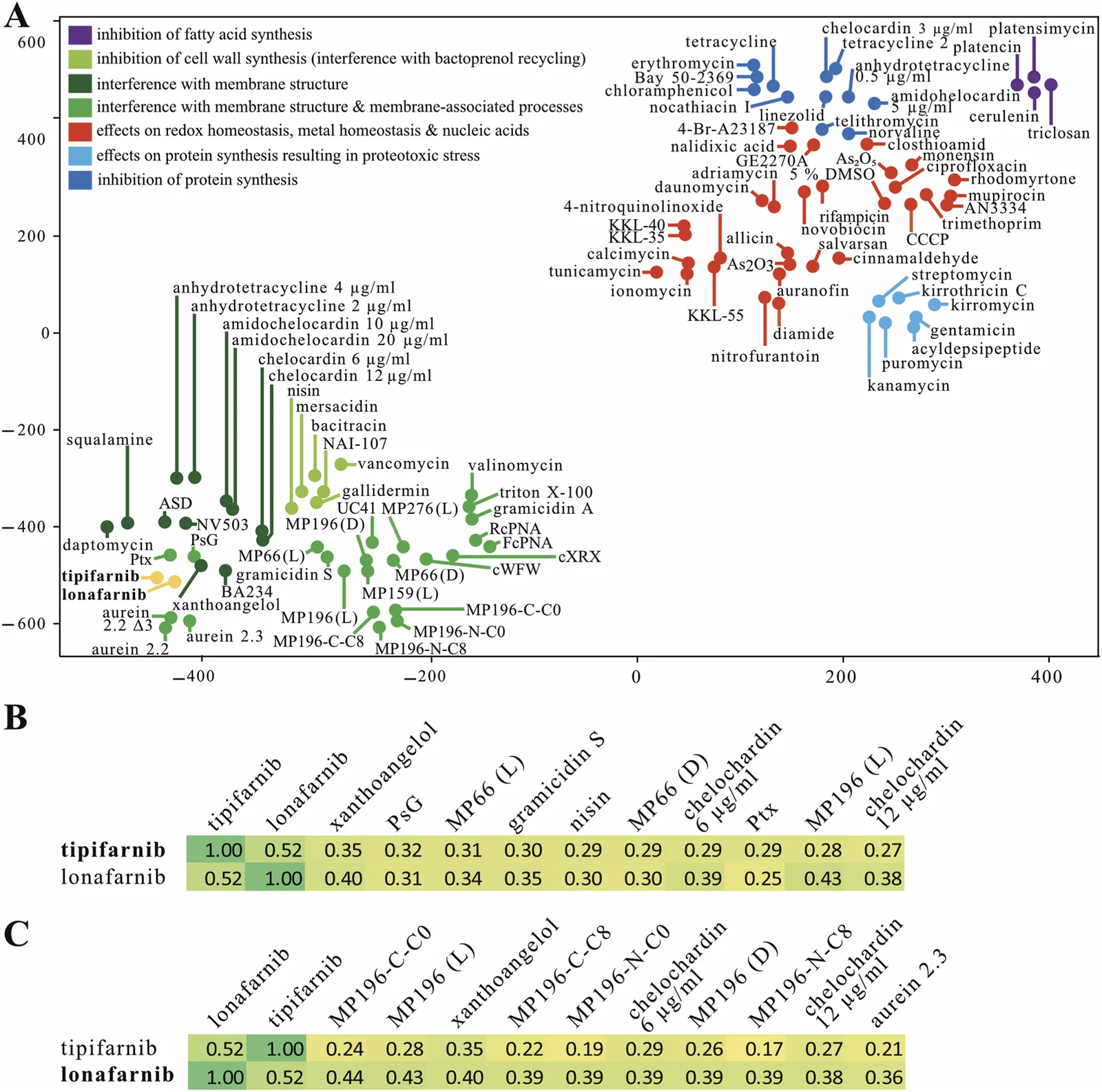
Comparative proteomic response (CoPR) analysis of farnesyltransferase inhibitors (FTIs) in *Bacillus subtilis*. 2D-PAGE-based proteomic responses of *Bacillus subtilis* to treatment with physiologically effective concentrations (PECs) of tipifarnib (7.5 μg/mL) or lonafarnib (12.5 μg/mL) (Figure 1) were compared with a reference library of 96 antibiotic agents, including many with known modes of action, as described earlier (Senges et al., 2020; Meier et al., 2019; Senges et al., 2022; Janzing et al., 2024). **(A)** Similarity between proteomic profiles was calculated using cosine similarity based on induction factors of marker proteins and visualized by t-distributed stochastic neighbor embedding (t-SNE). Each dot represents the proteomic profile of one reference antibiotic, color-coded by mode of action: membrane-active or inhibition of cell wall biosynthesis (greens), DNA/RNA synthesis inhibitors (red), protein synthesis inhibitors (blues) and fatty acid synthesis inhibitors (purple). Tipifarnib and lonafarnib are indicated by yellow lines highlighting their positions within the cluster of membrane-active and cell wall-targeting antibiotics. Heatmaps represent similarity scores between tipifarnib **(B)** and lonafarnib **(C)** and 10 other antibiotics with the highest similarity. High similarity is indicated by scores close to one and a high intensity of green color.

### 3.4 Proteomic response to FTI treatment assessed by gel-free LC-HDMS^E^

For proteomic profiling of accumulated proteins, bacterial cultures were grown to early log phase and treated with the physiologically effective concentrations for 60 min. Gel-free LC-HDMS^E^-based proteomic profiling was performed and proteins with log_2_ fold change outside the respective 95% confidence intervals and p < 0.05 were considered significantly regulated. Although the number of regulated proteins was similar, FTI treatment elicited markedly stronger proteomic changes in *B. subtilis* than in *E. coli* Δ*tolC*, with upregulated proteins in *B. subtilis* having higher induction factors. Additionally, tipifarnib affected the proteome of *E. coli* Δ*tolC* more significantly than lonafarnib, which only induced a very mild proteomic response reflected in comparably low induction factors.

In *B. subtilis*, tipifarnib treatment led to the regulation of 33 proteins, whereas lonafarnib treatment led to the regulation of 44 proteins (Figure 3; Table 3; Supplementary Figure S1). Tipifarnib treatment resulted in the regulation of proteins associated with the functional category “cell envelope and cell division” (YocH and YabE), as well as “coping with stress” (e.g., NfrA, LiaS, YugJ, and TrxA) (Figure 3A; Table 3; Supplementary Figure S1A). Lonafarnib treatment affected proteins associated with the cell wall (e.g., YocH, and YjoB) as well as several stress-related proteins (e.g., NfrA, YceC, and YvgN) (Figure 3B; Table 3; Supplementary Figure S1B). Interestingly, several regulated proteins were shared between tipifarnib and lonafarnib treatment (23 proteins in total, 0.43 similarity according to Jaccard-Index (Chung et al., 2019)). Many of these shared proteins were associated with the cell wall (e.g., YocH and YabE) as well as with the general stress response (e.g., SodA, Spx, TrxA, NfrA, and Hpf), including proteins regulated by the sigma factors σ^B^ (general stress response), σ^W^ (adaptation to membrane-active compounds, general cell envelope stress response), and σ^M^ (adaptation to inhibitors of peptidoglycan synthesis). This confirms that the antibacterial effect of FTIs in *B. subtilis* primarily involves the cell envelope.

**FIGURE 3:**
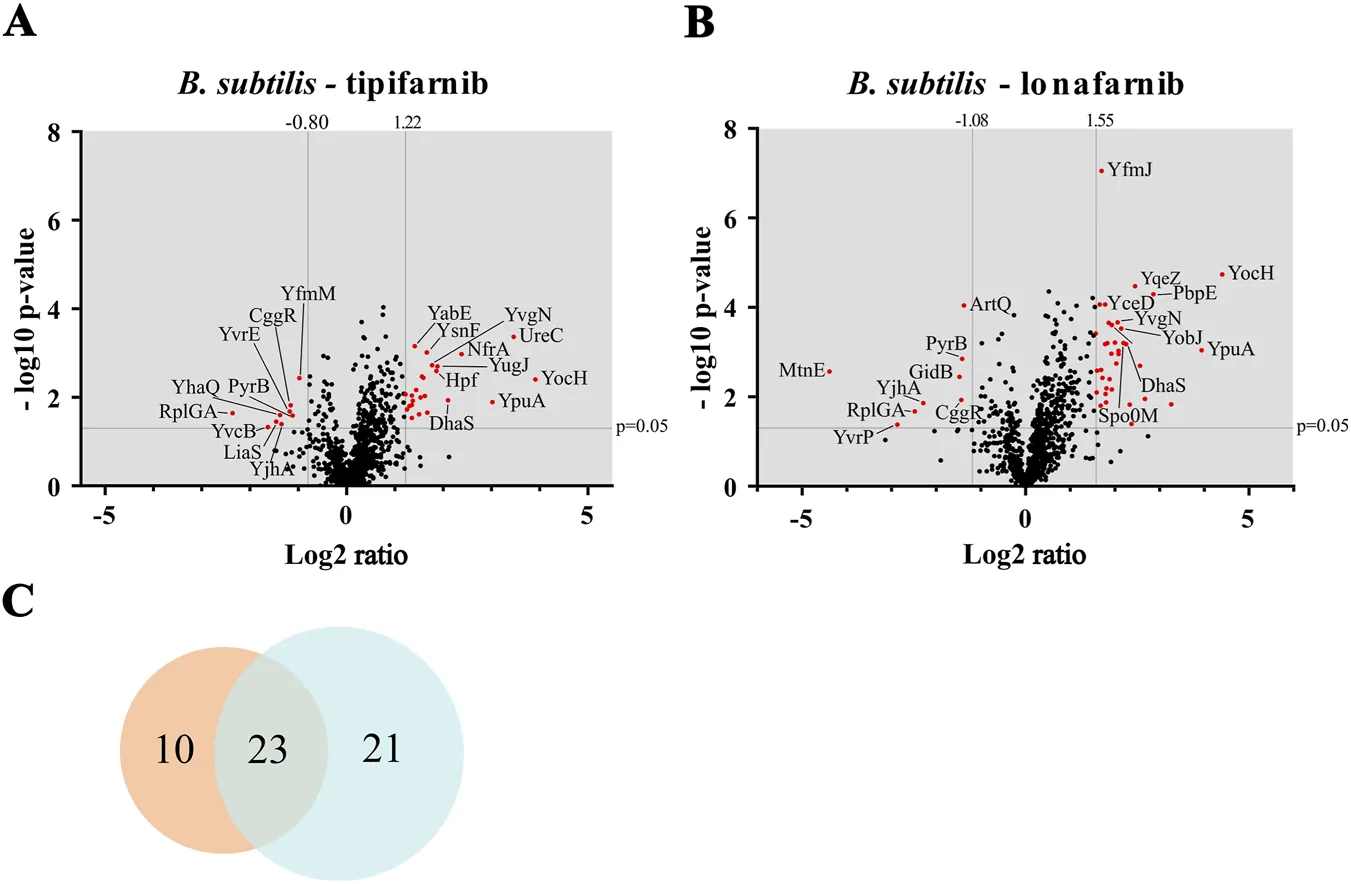
Proteomic response of *Bacillus subtilis* to farnesyltransferase inhibitors detected by gel-free LC-HDMS^E^ proteomics. Volcano plots show the protein abundance changes in *Bacillus subtilis* **(A,B)** after 60 min treatment with 7.5 μg/mL tipifarnib **(A)** or 12.5 μg/mL lonafarnib **(B)** compared to untreated controls. Proteins with log_2_ fold change outside the respective 95% confidence intervals and p < 0.05 were considered significantly regulated. Significantly regulated proteins are shown in red, non-significantly regulated proteins in black. The Venn diagram **(C)** shows the overlap between tipifarnib- (orange) and lonafarnib-regulated (blue) proteins. Number in intersection indicate number of proteins commonly regulated by both treatments. Numbers in non-overlapping areas indicate proteins uniquely regulated by each FTI. Experiments were performed as biological triplicates.

**TABLE 3:** Regulated proteins identified by gel-free proteomic profiling of *Bacillus subtilis* after 60 min tipifarnib or lonafarnib treatment. Proteins were categorized according to the Subtiwiki gene categories (level 2) (Elfmann et al., 2025). NR: not regulated. Proteins shown in bold were regulated in response to both tipifarnib and lonafarnib**.**

Protein	Category	Protein	Regulon	Tipifarnib		Lonafarnib	
				log2 ratio	p-value	log2 ratio	p-value
MtnE	Amino acid and nitrogen metabolism	Glutamine transaminase	​	NR		−4.39	0.0027
**UreC**		**Urease**	**PucR, GlnR, TnrA, CodY, σ^H^**	**3.46**	**0.0004**	**3.26**	**0.0148**
IolS	Carbon metabolism	Similar to dehydrogenase	IolR	1.25	0.0189	NR	
**CggR**		**Repressor of the glycolytic *gapA* operon**	**CggR**	**−1.16**	**0.0152**	**−1.44**	**0.0118**
**YabE**	Cell envelope and cell division	**Similar to cell wall binding protein**	​	**1.41**	**0.0007**	**1.86**	**0.0002**
FloT		Membrane-associated scaffold protein	σ^W^	NR		1.66	0.0001
PbpE		Class C penicillin-binding protein PBP 4*	σ^W^	NR		2.86	0.0001
YfnI		Polyglycerolphosphate lipoteichoic acid synthase	σ^M^	NR		1.8	0.0134
YjoB		Molecular chaperone	σ^W^	NR		1.6	0.0026
**YocH**		**Peptidoglycan hydrolase**	**Spo0A, WalR, AbrB**	**3.91**	**0.0040**	**4.4**	**0.0000**
**YpuA**	Coping with stress	**Unknown**	**σ^M^**	**3.02**	**0.0129**	**3.94**	**0.0009**
CopZ		Copper transport protein	CsoR	NR		1.68	0.0159
DisA		DNA integrity scanning protein	σ^M^, Spx, σ^B^, CtsR, σ^F^	NR		1.81	0.0065
**NfrA**		**Spx-dependent FMN-containing NADPH-linked nitro/flavin reductase**	**SpoOA, Spx, σ^D^**	**2.38**	**0.0011**	**2.67**	**0.0112**
YceD		General stress protein	σ^M^, σ^W^, σ^B^, σ^X^	NR		1.78	0.0001
RacX		Amino acid racemase	σ^W^	NR		2.0	0.0006
YdjO		Unknown	σ^E^, σ^W^	NR		2.33	0.0150
YknW		ABC transporter for the export of the SdpC toxin	σ^W^, AbrB	NR		1.83	0.0006
YobJ		Unknown	σ^W^	NR		2.14	0.0003
YqeZ		NfeD family protein NfeD1b	σ^W^	NR		2.45	0.0000
**Hpf**		**General stress protein**	**σ^B^, PhoP, σ^D^, σ^H^**	**1.86**	**0.0026**	**1.93**	**0.0069**
LiaS		Two-component sensor kinase/phosphatase	LiaR	−1.46	0.0356	NR	
**SodA**		**Superoxide dismutase**	**σ^B^**	**1.62**	**0.0093**	**1.72**	**0.0038**
**Spx**		**Transcriptional regulator spx**	**Spx**	**1.35**	**0.0293**	**1.88**	**0.0040**
**SwrC**		**Similar to acriflavin resistance protein**	​	**1.44**	**0.0068**	**1.57**	**0.0004**
**TrxA**		**Thioredoxin**	**Spx, σ^B^, CtsR**	**1.56**	**0.0034**	**1.69**	**0.0025**
TrxB		Thioredoxin reductase (NADPH)	Spx	1.22	0.0086	NR	
**YceC**		**Similar to tellurium resistance protein**	**σ^M^, σ^W^, σ^B^, σ^X^**	**1.5**	**0.0243**	**2.56**	**0.0020**
**YfhM**		General stress protein	**σ^W^, σ^B^**	**1.53**	**0.0101**	**2.08**	**0.0011**
**YjbC**		General stress protein	**PerR, σ^M^, σ^W^, σ^B^, σ^X^**	**1.37**	**0.0120**	**1.92**	**0.0011**
YsnF		General stress protein	σ^B^	1.66	0.0010	NR	
**YugJ**	​	**NADPH-dependent furan aldehyde reductase**	**Spx**	**1.88**	**0.0020**	**2.03**	**0.0018**
**YvgN**		**Glyoxal reductase**	**σ^B^, SinR**	**1.77**	**0.0019**	**2.06**	**0.0002**
YvrE		Glucono-lactonase	σ^B^	−1.18	0.0210	NR	
YhaQ	Membrane proteins	Similar to ABC transporter	AbrB	−1.38	0.0253	NR	
**YjhA**		**Lipoprotein**	​	**−1.35**	**0.0405**	**−2.29**	**0.0139**
**PyrB**	Nucleotide metabolism	**Aspartate carbamoyltransferase**	​	**−1.12**	**0.0259**	**−1.42**	**0.0014**
RadA		Branch migration transferase	σ^M^, Spx, σ^B^, CtsR, σ^F^	NR		1.78	0.0007
LipA	Other pathways	Lipoyl synthase	​	NR		1.59	0.0080
LplJ		Lipoate-protein ligase	​	1.3	0.0158	NR	
BstD	Phosphoproteins	Bacillithiol S-transferase	​	1.35	0.0150	NR	
**DhaS**	Poorly characterized	**Hydroxypropionaldehyde-specific aldehyde dehydrogenase (NAD)**	​	**2.1**	**0.0117**	**2.25**	**0.0007**
**YfmJ**		**Similar to predicted oxidoreductase**	**Spo0A, AbrB**	**1.35**	**0.0092**	**1.70**	**0.0000**
GidB	Protein homeostasis	Glucose-inhibited division protein	ComK, DnaA	NR		−1.48	0.0036
**RplGA**		Similar to ribosomal protein	σ^B^, NusA	**−2.36**	**0.0229**	**−2.48**	**0.0213**
YfmM		Translation factor	​	−0.98	0.0037	NR	
Hit	Regulation of gene expression	Hit-like protein involved in cell-cycle regulation	​	NR		2.37	0.0406
Spo0M	Sporulation	Sporulation-control gene	σ^W^, σ^H^	NR		2.19	0.0006
ArtQ	Transporters	High affinity arginine ABC transporter	RnpM	NR		−1.38	0.0001
**YqiG**	Unknown	**Similar to NADH-dependent flavin oxidoreductase**	​	**1.67**	**0.0223**	**2.08**	**0.0009**
**YrrB**		**Unknown**	​	**1.59**	**0.0036**	**1.93**	**0.0002**
YvcB		Unknown	DegU, AbrB	−1.63	0.0473	NR	
YwjG		Unknown	​	NR		1.79	0.0087
YvrP		Putative efflux system protein	ComK, AbrB	NR		−2.87	0.0421

Bold indicates proteins regulated in response to both tipifarnib and lonafarnib.

YceC, YvgN and TrxA were detected in the 2D-PAGE-based analysis as well as in the gel-free LC-HDMS^E^ dataset upon both treatments (Table 2; Table 3), suggesting that these proteins remain upregulated beyond the immediate stress response phase and accumulate over time. The same applies to YpuA, YceD, SodA and Spo0M under lonafarnib treatment and to YugJ and BstD under tipifarnib treatment (Table 2; Table 3).

In *E. coli* Δ*tolC*, tipifarnib treatment resulted in the regulation of 42 proteins, whereas lonafarnib induced the regulation of 20 proteins (Figure 4A; Table 4; Supplementary Figure S2A). Based on GO terms (Ashburner et al., 2000; Consortium, 2026), the cellular distribution of tipifarnib-regulated proteins showed that 20 proteins (e.g., Tdk, RaiA, UspA or SrkA) localize to the cytoplasm, while 21 proteins were assigned to the GO categories “outer membrane”, “outer membrane-bounded periplasmic space”, “plasma membrane”, “periplasm” and “periplasm, secreted” (Table 4; Supplementary Figure S2A). The category “outer membrane-bounded periplasmic space” included, for example, the proteins PotF, CpxP, DsbC, PpiA, OsmY, MppA, and MglB, and was found to have an enrichment factor of 6.62 for tipifarnib using the PANTHER overrepresentation test (Mi et al., 2013), while no other cellular component category was significantly enriched (Table 4; Supplementary Figure S2A).

**FIGURE 4:**
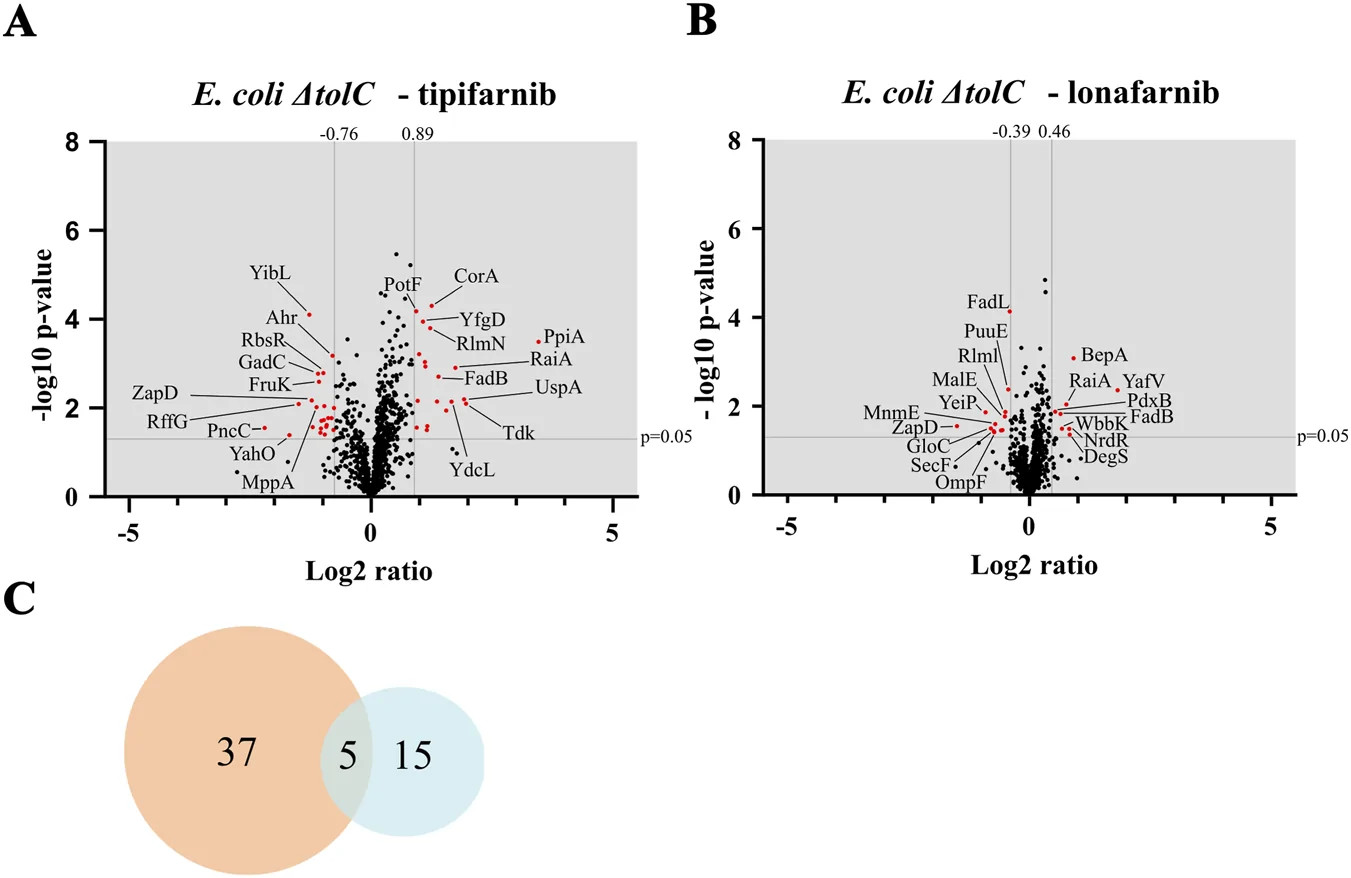
Proteomic response of *Escherichia coli* Δ*tolC* to farnesyltransferase inhibitors detected by gel-free LC-HDMS^E^-based proteomics. Volcano plots show the protein abundance changes in *Escherichia coli* Δ*tolC* **(A,B)** after 60 min treatment with 12.5 μg/mL tipifarnib **(A)** or 50 μg/mL lonafarnib **(B)** compared to untreated controls. Proteins with log_2_ fold changes outside the respective 95% confidence intervals and p < 0.05 were considered significantly regulated. Significantly regulated proteins are shown in red, non-significantly regulated proteins in black. The Venn diagram **(C)** shows the overlap between tipifarnib- (orange) and lonafarnib-regulated (blue) proteins. Number in intersection indicate number of proteins commonly regulated by both treatments. Numbers in non-overlapping areas indicate proteins uniquely regulated by each FTI. Experiments were performed as biological triplicates.

**TABLE 4:** Regulated proteins identified by gel-free proteomic profiling in *Escherichia coli* Δ*tolC* after tipifarnib or lonafarnib treatment for 60 min. Proteins were categorized according to the GO terms for cellular components (Ashburner et al., 2000; Consortium, 2026). NR: not regulated. Proteins shown in bold were regulated in response to both tipifarnib and lonafarnib.

Protein	Cellular localization	Protein function	Tipifarnib		Lonafarnib	
			log2 ratio	p-value	log2 ratio	p- value
Cfa	Cytoplasm	Cyclopropane-fatty-acyl-phospholipid synthase (membrane biosynthesis)	−1	0.0196	NR	
DhaK		Phosphoenolpyruvate-dependent dihydroxyacetone kinase	−0.9	0.0266	NR	
**FadB**		**Fatty acid oxidation complex subunit alpha**	**1.4**	**0.0020**	**0.6**	**0.015**
FolC		Dihydrofolate synthase	1.2	0.0257	NR	
FruK		1-Phosphofructokinase	−1.1	0.0026	NR	
FumB		Fumarate hydratase class I, anaerobic	0.9	0.0278	NR	
GloC		Hydroxyacylglutathione hydrolase	NR		−0.8	0.0318
MnmE		tRNA modification GTPase	NR		−0.7	0.0253
MsrB		Peptide methionine sulfoxide reductase	1	0.0069	NR	
NrdR		Transcriptional repressor	NR		0.8	0.0326
PdxB		Erythronate-4-phosphate dehydrogenase	NR		0.5	0.0133
PuuE		4-Aminobutyrate aminotransferase	NR		−0.4	0.0042
**RaiA**		**Ribosome-associated inhibitor A**	**1.7**	**0.0012**	**0.8**	**0.0092**
RlmI		Ribosomal RNA large subunit methyltransferase I	NR		−0.5	0.0135
RlmN		Dual-specificity RNA methyltransferase	1.2	0.0002	NR	
RpmB		Large ribosomal subunit protein bL28	NR		−0.6	0.0353
SrkA		Stress response kinase A	1.5	0.0115	NR	
TabA		Toxin-antitoxin biofilm protein	−1	0.0290	NR	
Tdk		Thymidine kinase	2	0.0080	NR	
UspA		Universal stress protein A	1.9	0.0064	NR	
YeiP		Elongation factor protein	NR		−0.9	0.0138
YfgD		Uncharacterized protein	1.1	0.0001	NR	NR
**ZapD**		**Cell division protein**	**−1.2**	**0.0068**	**−1.5**	**0.0282**
Ahr		Aldehyde reductase	−0.8	0.0007	NR	
PncC		Nicotinamide-nucleotide amidohydrolase	−2.2	0.0283	NR	
RbsR		Ribose operon repressor	−1	0.0016	NR	
RffG		dTDP-glucose 4,6-dehydratase 2	−1.5	0.0082	NR	
YafV		Omega-amidase	NR		1.8	0.0044
YibL		Unknown function	−1.3	0.0001	NR	
BepA	Outer membrane-bounded periplasmic Space	Beta-barrel assembly-enhancing protease	NR		0.9	0.0008
CpxP		Periplasmic protein	1	0.0006	NR	
DegS		Serine endoprotease	NR		0.8	0.044
DsbC		thiol:disulfide interchange protein	1.1	0.0009	NR	
Eco		Ecotin	−0.9	0.0169	NR	
Ivy		Inhibitor of vertebrate lysozyme	−0.8	0.0314	NR	
LivJ		Leu/Ile/Val-binding protein	−0.9	0.0243	NR	
**MalE**		**Maltose/maltodextrin-binding periplasmic protein**	**−0.8**	**0.017**	**−0.5**	**0.0171**
MglB		D-galactose/methyl-galactoside binding periplasmic protein	−1	0.0398	NR	
MppA		Periplasmic murein peptide-binding protein	−1.1	0.0096	NR	
OsmY		Osmotically-inducible protein	−1.2	0.0271	NR	
PliG		Inhibitor of g-type lysozyme	−1	0.0091	NR	
PotF		Putrescine-binding periplasmic protein	0.9	0.0001	NR	
PpiA		Peptidyl-prolyl cis-trans isomerase A	3.5	0.0003	NR	
SecF		Protein translocase subunit	NR		−0.7	0.0366
TcyJ		L-cystine-binding protein	−0.8	0.0101	NR	
CusC	Outer Membrane	Cation efflux system protein	1.2	0.0316	NR	
FadL		Long-chain fatty acid transport protein	NR		−0.4	0.0001
OmpF		Outer membrane porin F	NR		−0.7	0.0387
OmpN		Outer membrane porin N	NR		−0.6	0.0346
YahO	Periplasm	Uncharacterized protein	−1.7	0.041	NR	
YebF	Periplasm, secreted	Unknown function	−1	0.0188	NR	
CorA	Plasma Membrane	Magnesium transport protein	1.3	0.0001	NR	
GadC		Glutamate/gamma-aminobutyrate antiporter	−1.1	0.0017	NR	
NuoE		NADH-quinone oxidoreductase subunit E	−1	0.0363	NR	
**WbbK**		**Putative glycosyltransferase**	**1.1**	**0.0012**	**0.7**	**0.0323**
YdcL		Uncharacterized lipoprotein	1.7	0.0072	NR	
YibT	Unknown localization	Unknown function	1.4	0.0072	NR	

Bold indicates proteins regulated in response to both tipifarnib and lonafarnib.

Lonafarnib affected a smaller set of 20 proteins with only low induction factors (Figure 4B; Table 4; Supplementary Figure S2B). However, the regulation of membrane-associated proteins (FadL, OmpN, and OmpF) and proteins involved in membrane-bound transport processes (MalE and SecF) were also observed. Interestingly, a subset of proteins was commonly regulated in response to both FTIs (FadB, ZapD, WbbK, MalE, RaiA) corresponding to an overlap of 16% (Table 4).

### 3.5 Membrane integrity and membrane potential (ΔΨ) assessment confirms proteomics-based predictions

As both proteomic approaches indicate that FTIs affect the bacterial membrane in both organisms, membrane integrity was assessed using SYTO9 and propidium iodide (PI) staining. While SYTO9 stains all cells, propidium iodide only stains cells with impaired membrane integrity. Nisin at 20 μg/mL for *B. subtilis* and colistin at 2 μg/mL for both *E. coli* strains served as membrane-permeabilizing positive controls and produced the expected PI-positive response, thereby confirming assay responsiveness under the respective experimental conditions. In *B. subtilis,* treatment with tipifarnib (7.5 and 10 μg/mL) and lonafarnib (12.5 and 25 μg/mL) resulted in substantial cytoplasmic membrane damage, indicated by propidium iodide staining (Figure 5A). The lower concentrations (7.5 μg/mL tipifarnib and 12.5 μg/mL lonafarnib) resulted in a lower propidium iodide signal, indicating that the response is dose-dependent (Figure 5C). In *E. coli*, propidium iodide uptake was limited to the Δ*tolC* strain (Figures 5B,D). The wild-type strain only showed a slight increase in membrane permeability when exposed to a very high lonafarnib concentration of 100 μg/mL.

**FIGURE 5:**
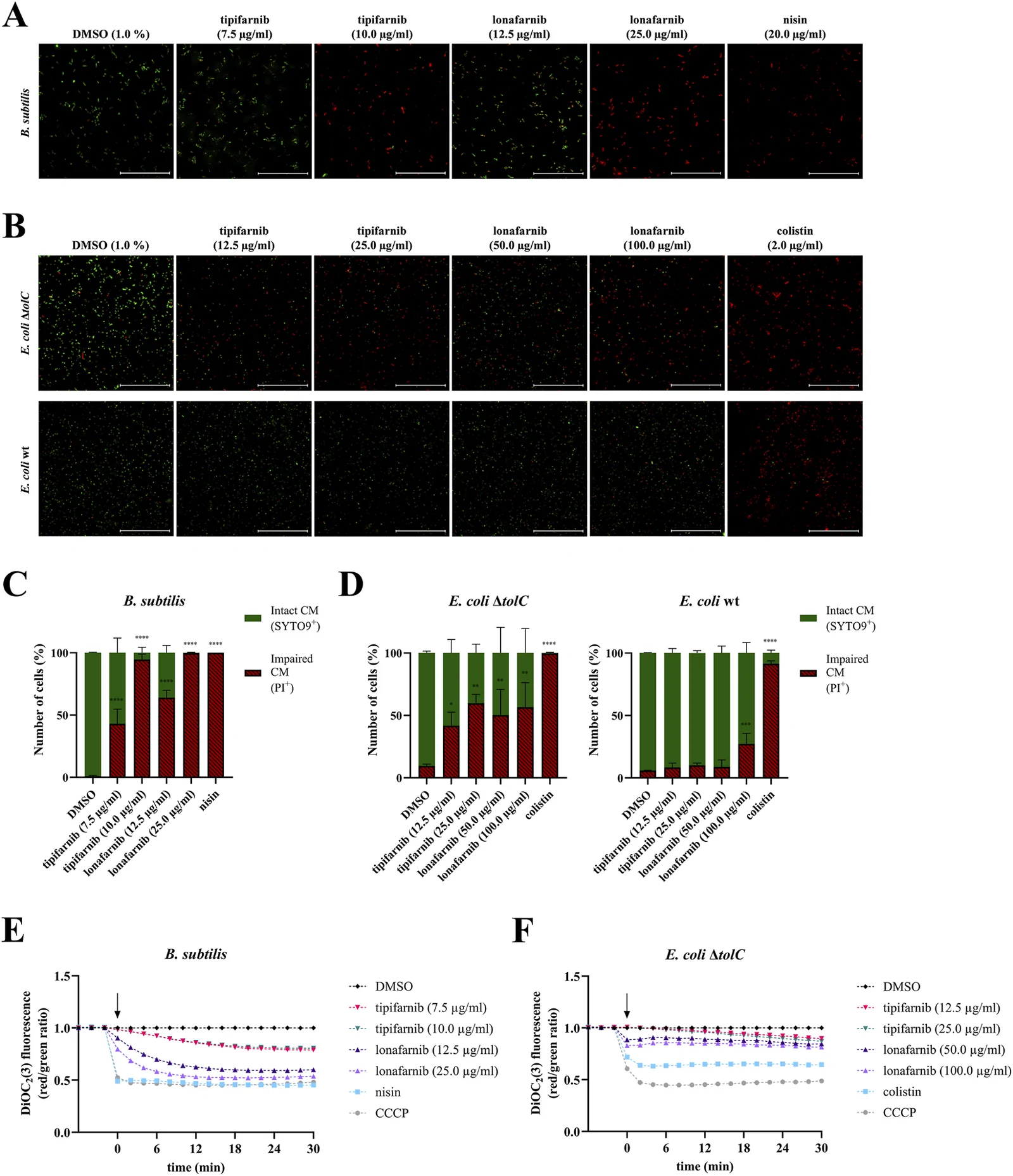
Membrane integrity and membrane potential of *Bacillus subtilis* and *Escherichia coli* after FTI treatment. **(A,B)** Cytoplasmic-membrane permeability assessed with SYTO9 (membrane-permeable, green) and propidium iodide (PI, membrane-impermeant, red). Representative micrographs from three biological replicates are shown for **(A)** *Bacillus subtilis* and **(B)** *Escherichia coli* wt (wild type) and Δ*tolC*. Tipifarnib was applied at concentrations of 7.5 μg/mL and 10 μg/mL in *Bacillus subtilis*, and 12.5 μg/mL and 25 μg/mL in *Escherichia coli* wt and Δ*tolC*. Lonafarnib was applied at concentrations of 12.5 μg/mL and 25 μg/mL in *Bacillus subtilis*, and 50 μg/mL and 100 μg/mL in *Escherichia coli* wt and Δ*tolC*. Colistin (2 μg/mL), nisin (20 μg/mL), and DMSO (1%) served as controls. Scale bars: 100 µM. **(C,D)** Quantification of SYTO9/propidium iodide staining from microscopy images, reported as the red-to-green fluorescence ratio. Three independent experiments were quantified with ≥100 cells analyzed per condition; data are shown as means ± SD **(E,F)** Membrane potential was assessed using 30 µM DiOC_2_ (Fiordalisi et al., 2003) in **(E)** *Bacillus subtilis* and **(F)** *Escherichia coli* Δ*tolC*. DMSO (1%) served as negative control, whereas CCCP (5 µM), nisin (20 μg/mL) and colistin (2 μg/mL) were used as positive controls. Baseline membrane potential was recorded prior to inhibitor addition at t = 0 min. The arrow shows the first recorded measurement after compound addition. * Indicates p ≤ 0.05, ** indicates p < 0.01, *** indicates p < 0.001, **** indicates p < 0.0001.

Additionally, a membrane potential assay based on DiOC_2_ (Fiordalisi et al., 2003) was performed in order to further validate one component of the proton-motive force (Figures 5E,F). Here, carbonyl cyanide 3-chlorophenylhydrazone (CCCP), a proton ionophore, served as a positive control at 5 µM and induced membrane depolarization. Similar to the membrane integrity assay, nisin (*B. subtilis*) and colistin (*E. coli* Δ*tolC*) served as controls and caused a decrease in the red/green fluorescence ratio, indicating loss of membrane potential. In *B. subtilis*, both tipifarnib and lonafarnib decreased the membrane potential, with lonafarnib showing a more pronounced and dose-dependent effect (Figure 5E). In *E. coli* Δ*tolC*, the effect was less pronounced, although a decrease was also observed (Figure 5F).

Since effects on membrane integrity and potential often compromise cellular metabolic homeostasis and trigger oxidative stress, we additionally evaluated the effects on the intracellular redox state using DCFH-DA (Supplementary Figure S3). Here, treatment with tipifarnib induced a significant shift in the intracellular redox state after 1 hour, as indicated by an increase in DCF fluorescence intensity. The treatment with lonafarnib had a more limited impact on cellular oxidation under these conditions, showing a significant shift only with 12.5 μg/mL lonafarnib in *B. subtilis* and no effect in *E. coli* Δ*tolC* (Supplementary Figure S3).

## 4 Discussion

This study addresses the antibacterial mode of action of the FTIs tipifarnib and lonafarnib against the Gram-positive model organism *B. subtilis* and the efflux-deficient Gram-negative strain *E. coli* Δ*tolC*. The efficacy of FTIs against *S. aureus*, MRSA, *S. epidermidis,* and *S. pneumonia*e has already been shown (Weber et al., 2021). Our current work confirms this activity in the well-established model organism *B. subtilis*, which is frequently used in mode of action studies. While Gram-negative bacteria are typically intrinsically resistant to FTIs due to their cell envelope architecture, we previously demonstrated that sub-inhibitory concentrations of colistin can sensitize Gram-negative ESKAPE pathogens to these agents by permeabilizing the outer membrane (Klose et al., 2025). To investigate the mode of action without the use of permeabilizers, we employed an *E. coli* Δ*tolC* strain, which is efflux deficient but is also known to have pleiotropic defects, especially regarding outer membrane composition (Wright et al., 2025). Compared to the parental *E. coli* strain, the Δ*tolC* strain was sensitive to moderate amounts of FTIs. While the relevance of this model to wildtype Gram-negative pathogens remains limited due to artificial sensitization, it nevertheless enables mechanistic insights into the mode of action of FTIs in a Gram-negative background. Our study integrates susceptibility testing, comparative proteomic response profiling, as well as membrane integrity and membrane potential assays to support the proteomic profiling results.

MIC testing and determination of physiologically effective concentrations revealed a strain-specific susceptibility pattern, with *B. subtilis* being two to four-fold more sensitive than *E. coli* Δ*tolC.* Tipifarnib was consistently more effective than lonafarnib in both organisms, a difference that likely reflects variations in uptake or target affinity (Bos et al., 2007; End et al., 2001). This difference in potency did not affect the examination of the proteomic responses, as compound-specific physiologically effective concentrations were used to ensure comparability across all proteomic analyses.

The proteomic response of *B. subtilis* towards tipifarnib and lonafarnib was initially analyzed using a 2D-PAGE approach to monitor the differential abundance of newly synthesized proteins labeled with [^35^S]-methionine upon short-time exposure, comparing the results to an established reference library. However, as no such reference library is available for *E. coli*, we monitored changes in the accumulated proteome of *E. coli* Δ*tolC* after 60 min of treatment by gel-free proteomics. This method provides insights into long-term adaptations and can capture a broader range of proteins, including those that are challenging to resolve using 2D-PAGE. To facilitate comparative analysis and ensure comprehensive data, we applied the same gel-free proteomics method to *B. subtilis*. For *B. subtilis* it can be additionally advantageous to apply both techniques if activation of the general stress response was monitored with newly synthesized proteins in the acute stress response. The general stress response is transiently activated, peaking 10–40 min following an acute stress with a rapid decline of SigB activity thereafter (Voelker et al., 1995). Monitoring the accumulated proteome after 60 min of stress can therefore help to further derive sustained or delayed cellular adaptations indicative of the mode of action.

In *B. subtilis*, both responses were dominated by proteins involved in cell envelope stress, peptidoglycan turnover, and general stress adaptation, a profile characteristic of antibiotics targeting the cell envelope (Senges et al., 2020). This included the upregulation of LiaH in the 2D-PAGE analyses, which acts as a sensitive marker of cell envelope stress (Wolf et al., 2010; Schrecke et al., 2013; Bernal-Cabas et al., 2020) and can be upregulated in response to inhibition of cell wall biosynthesis (bactoprenol-cycle) as well as disturbance of structural membrane integrity and membrane-associated functions (Senges et al., 2020). Furthermore, activation of the σ^M^-regulon, a hallmark of cell envelope stress (Eiamphungporn and Helmann, 2008), led to the induction of YpuA (2D-PAGE) and the lipoteichoic acid synthase YfnI/LtaS (gel-free) upon lonafarnib treatment. Lipoteichoic acids are essential components of the Gram-positive cell wall and are critical for membrane integrity (Garufi et al., 2012; Percy and Grundling, 2014; Hashimoto et al., 2013). Activation of the σ^W^-regulon, responding to cell envelope damage and allowing adaptation to membrane-active compounds (Helmann, 2016; Cao et al., 2002), was further supported by the induction of FloT (Bach and Bramkamp, 2013; Yepes et al., 2012) and the penicillin-binding protein PbpE (Cao et al., 2002; Popham and Setlow, 1993). The strong upregulation of the peptidoglycan hydrolase YocH under both treatments may indicate increased cell wall remodeling and repair (Vollmer, 2012; Hayhurst et al., 2008; Brogan and Rudner). The strong induction of the phage shock protein A (PspA) under both treatments, which helps to maintain membrane integrity, provides evidence that FTIs impair membrane integrity (Scholz et al., 2021).

It is known that membrane-active antibiotics can induce the general stress response, and accordingly, many upregulated proteins are encoded by σ^B^-regulated genes (Senges et al., 2020). Additionally, tipifarnib and lonafarnib also led to induction of proteins encoded by Spx-regulated genes in both the early and late stress response, which can be activated *via* the σ^B^, σ^M^ and σ^W^ regulons, all of which appear to be active upon FTI treatment (Eiamphungporn and Helmann, 2008; Tam le et al., 2006). This suggests that FTIs cause oxidative stress.

Comparing the proteomic profiles of lonafarnib and tipifarnib using the 2D-PAGE approach to the reference library revealed that they clustered with those of membrane-active antibiotics. The similarity to antimicrobial peptides like MP196, Ptx, and PsG suggests that FTIs induce a proteomic stress signature characteristic of compounds that impair membrane function and may indirectly affect cell wall biosynthesis (Meier et al., 2019; Janzing et al., 2024; Senges et al., 2020; Straus and Hancock, 2006). Also, both FTIs showed similarity to nisin, which binds the cell wall precursor lipid II and forms pores, thereby disrupting membrane integrity and inhibiting cell wall biosynthesis. Notably, both FTIs exhibited the highest similarity to each other within the CoPR analysis, while only a lower similarity to other proteomic profiles was observed. This consistent clustering of the FTIs suggests a shared global mode of action with membrane-active and possibly indirectly cell-wall-targeting antibiotics but a molecular mode of action that is not represented within the current reference set. In this context, such a mode of action could be of particular interest regarding antimicrobial resistance, as it may reduce the likelihood of cross-resistance with established antibiotic classes.

In *E. coli* Δ*tolC,* the lower susceptibility towards lonafarnib was reflected in a comparatively mild proteomic response, with very low induction factors for regulated proteins and only two of the 20 proteins being up- or downregulated at least two-fold (YafV, ZapD). Interestingly, lonafarnib treatment led to the downregulation of porins such as OmpN and OmpF, which may be consistent with reduced compound uptake and the higher MIC compared to tipifarnib (Duval et al., 2009).

Analysis of the subcellular localization of proteins regulated in response to tipifarnib in *E. coli* Δ*tolC* indicated changes predominantly affecting proteins in the periplasm, as well as proteins located in the inner and outer membrane. With WbbK and RffG being regulated, effects on membrane integrity might be counteracted by modulating lipopolysaccharide and enterobacterial common antigen biosynthesis (Martorana et al., 2014; Meier-Dieter et al., 1992). Notably, tipifarnib treatment led to the upregulation of five proteins regulated by the Cpx envelope stress response system (SrkA, RaiA, CpxP, DsbC, PpiA). The Cpx envelope stress response is activated by different stressors including aminoglycoside antibiotics, acid stress, and alkaline stress, which are thought to lead to the accumulation of misfolded or mislocalized cell envelope proteins (Kohanski et al., 2008). In line with this, NuoE was downregulated, which is encoded by the *nuo* operon that is repressed by Cpx in response to envelope stress (Guest et al., 2017). Additionally, cells might encounter oxidative stress as MsrB, which rescues oxidized methionine in peptides as well as RlmN, which is activated by ROS and improves translational efficiency and fidelity (Benítez-Páez et al., 2012), were upregulated. This might explain the upregulation of other proteins that contain iron-sulfur clusters (FumB) or bind divalent cations, such as FolC and Tdk, which might be damaged by oxidative stress. In line with this, the upregulation of a transport system for Mg^2+^/Co^2+^/Mn^2+^ (CorA), together with the downregulation of FruK, might represent a coping strategy to reduce the burden of ROS generation in *E. coli,* as discussed previously (Metwally and Ikeno, 2026). This was further supported by measurements of the intracellular oxidation of DCFH-DA to assess the cellular redox state, which indicated oxidative stress caused by tipifarnib treatment in both *B. subtilis* and *E. coli* Δ*tolC*. In contrast, lonafarnib altered the cellular oxidation state in *B. subtilis* only at the concentration used for proteomic profiling, whereas no such effect was observed in *E. coli* Δ*tolC* (Supplementary Figure S3).

As bacteria do not possess the eukaryotic target farnesyltransferase, alternative mechanisms must be considered. It cannot be excluded that FTIs could have a protein target in bacteria, which needs to be addressed in the future. We show that FTIs cause a proteomic response in both *B. subtilis* and *E. coli* Δ*tolC* that is indicative of a pronounced impairment of membrane barrier function. This is supported by the impaired membrane integrity monitored by the uptake of propidium iodide as well as the decrease in the membrane potential upon FTI treatment, which is more pronounced in *B. subtilis* under lonafarnib treatment. FTIs might have a dual mode of action, similar to atypical tetracyclines, which inhibit protein biosynthesis at lower concentrations but target the bacterial membrane at higher concentrations (Stepanek et al., 2016). Alternatively, FTIs may act primarily on the bacterial cell envelope. Our data suggest that the cytoplasmic membrane might be the primary target. However, in *B. subtilis*, proteins belonging to the σ^M^ regulon, which responds to inhibitors of peptidoglycan synthesis, were also upregulated in both proteomic approaches, indicating that this pathway may be affected either directly or indirectly through perturbation of membrane-bound steps of cell wall biosynthesis.

For *E. coli*, our results need to be interpreted in the context of the *tolC* knockout*,* which increases susceptibility to many antibiotics due to decreased efflux and has also been shown to cause pleiotropic effects, including outer membrane defects (Fralick, 1996; Zhu et al., 2024). Whether disturbance of the outer membrane function of *E. coli* Δ*tolC* or decreased efflux cause increased sensitivity towards FTIs needs to be investigated further. We have shown previously that co-treatment with sub-inhibitory concentrations of colistin, which primarily disrupts the outer and subsequently the inner membrane of Gram-negative bacteria, sensitizes *E. coli* and other Gram-negative bacteria to FTI treatment (Klose et al., 2025). These findings suggest that Gram-negative bacteria could potentially be targeted by optimizing FTIs for improved outer-membrane penetration or by combining them with appropriate sensitizing agents.

In subsequent studies, effects on the cell wall of *B. subtilis* should be further substantiated. Furthermore, it should be analyzed whether these effects are caused directly or indirectly by the perturbation of membrane-bound steps in cell wall biosynthesis. Overall, FTIs seem to target the cell envelope in both Gram-negative and Gram-positive bacteria.

While the observed antibacterial activity of FTIs supports their potential for drug repurposing, their known activity in eukaryotic cells may limit their direct application as anti-infective agents in standard clinical settings. In particular, host-directed effects could lead to off-target interactions that restrict their use in uncomplicated infections. However, some of their host-directed effects, such as immunomodulatory properties, may be particularly beneficial in specific clinical contexts, especially in severe or systemic infections.

In this context, FTIs may be more suitable as lead structures for the development of novel antibacterial compounds with reduced activity against eukaryotic targets. For lonafarnib, attempts are already being made to reduce the toxicity of lonafarnib to nonmalignant cells by means of chemical modifications (Hongnak and Gust, 2023). Additionally, FTIs have so far been tested primarily for their tolerability during long-term use, for example, as part of chemotherapy or as a daily treatment for progeria (lonafarnib). The side-effect profile of non-accumulating doses - for example, in a short-term treatment of a severe infection - may therefore be completely different (Awada et al., 2002; Perez-Ruixo et al., 2006). Furthermore, FTIs do not necessarily have to be used as monotherapy; a reduction in dosage through combination with colistin has already been demonstrated for several ESKAPE pathogens (Klose et al., 2025), and it is conceivable that further synergistic potential with other antibiotics could be exploited. Consequently, a certain degree of cytotoxicity is not a categorical exclusion criterion. This is exemplified by polymyxins, which exhibit significant nephrotoxicity yet remain in clinical use following a careful risk-benefit assessment (Wagenlehner et al., 2021).

## 5 Conclusion

Our complementary proteomic approaches demonstrate that FTIs induce a proteomic response consistent with cell envelope perturbation, most likely based on disturbed membrane integrity, with pronounced strain-specific differences. CoPR analysis showed that FTIs cluster with membrane-active antibiotics as well as antibiotics targeting membrane-bound steps of cell wall biosynthesis. Yet, the proteomic profiles are not identical, indicating a related but distinct mode of action. Further studies are required to identify the molecular target and to explore structure-activity relationships, with the aim of developing antibacterial agents based on FTIs as lead structures. In addition, their potential dual activity, combining direct antibacterial effects with immunomodulatory properties, warrants further investigation in the context of severe infections.

## Data Availability

The mass spectrometry proteomics data have been deposited to the ProteomeXchange Consortium via the PRIDE (Perez-Riverol et al., 2025) partner repository with the dataset identifier PXD081045.
